# Post-COVID syndrome patients show reduced anti-Spike antibodies compared to COVID-recovered controls, but enhanced IgG4/IgG1 switch after the third vaccine dose

**DOI:** 10.3389/fimmu.2025.1670324

**Published:** 2025-10-02

**Authors:** Nineth Rossi, Javier Benítez-Cruz, Patricia Marín-García, Isabel G. Azcárate, Alba González-Escalada, Oscar G. Hervás, Balbino Alarcón, José R. Regueiro, José M. Bautista, Narcisa Martinez-Quiles

**Affiliations:** ^1^ Department of Immunology, Ophthalmology and ENT, Complutense University School of Medicine, Madrid, Spain; ^2^ Immunology Section, Fac. de CC. de la Salud, Departamento de Especialidades Médicas y Salud Pública, Universidad Rey Juan Carlos (URJC), Alcorcón, Spain; ^3^ Microbiology Section, Fac. de CC. de la Salud, Departamento de Especialidades Médicas y Salud Pública, Universidad Rey Juan Carlos (URJC), Alcorcón, Spain; ^4^ Immune System Development and Function Program, Centro Biología Molecular Severo Ochoa, Consejo Superior de Investigaciones Científicas, Universidad Autónoma de Madrid, Cantoblanco, Spain; ^5^ Research Institute Hospital 12 de Octubre (imas12), Madrid, Spain; ^6^ Department of Biochemistry and Molecular Biology, Universidad Complutense de Madrid, Madrid, Spain; ^7^ Area of Infectious Diseases and AIDS, Research Institute Hospital 12 de Octubre (imas12), Madrid, Spain

**Keywords:** SARS-CoV-2, post-COVID, antibody response, IgG subclass, IgG4, Spike protein, vaccination, reinfection

## Abstract

**Introduction:**

Long COVID and post-COVID syndromes represent a significant global health crisis and a substantial societal challenge. Although an altered immunological response has been suggested as a possible underlying mechanism, the antibody response to vaccination and infection of the patients remains unclear.

**Methods:**

We studied a post-COVID syndrome cohort compared to a COVID-recovered cohort. Initially, we established the risk factors and the evolution of symptoms. Then, we analyzed the antibody response, focusing on immunoglobulin subclasses. Apart from determining immunoglobulin G (IgG) against the Nucleocapsid, which is a marker of infection, we analyzed IgG and its subclasses against the full-length Spike, and against the receptor-binding domain (RBD). Additionally, we examined the switch to IgG4, which can be promoted by repeated antigen exposure.

**Results:**

We show the major risk factors for developing post-COVID syndrome, such as infection before vaccination and comorbidities. Furthermore, we describe the evolution of the post-COVID symptoms, which agrees with previous reports. Regarding the antibody response, we found that compared to COVID-recovered individuals, post-COVID patients present readily detectable anti-Nucleocapsid IgG but low quantities of anti-Spike antibodies. Nevertheless, the anti-RBD IgG1 levels are similar between post-COVID and COVID samples. Interestingly, post-COVID patients with three vaccine doses, who were infected before vaccination by the Wuhan strain and subsequently reinfected post-Omicron, show decreased Spike response but intensified anti-RBD IgG4/IgG1 switch, compared to their non-reinfected post-COVID counterparts.

**Discussion:**

Our results support a differential antibody response in post-COVID versus COVID-recovered patients, which might be relevant for post-COVID syndrome treatment, including appropriate recall vaccination strategies for the still-circulating SARS-CoV-2.

## Graphical Abstract

Study of the antibody response of a post-COVID syndrome cohort from Comunidad de Madrid (Spain). A post-COVID cohort (n= 104) and a COVID- recovered cohort (n= 30) were studied and compared. Risks factors were determined. Specific immunoglobulins against the Nucleocapsid, the full-length Spike and the receptor-binding domain (RBD) of SARS-CoV-2 were determined and analyzed. Image was produced with Adobe Photoshop CS6 using NIH Bioart illustrations.

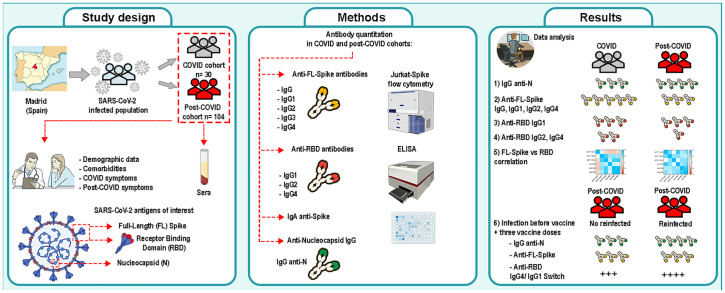

## 1 Introduction

Coronavirus disease 2019 (COVID-19) has caused a global health crisis. As of 2023, more than 700 million COVID-19 cases were reported worldwide during the pandemic period, and at least 6%, with a maximum estimated at 21% of infections in adults, and 1% in children could have led to the development of diverse health sequelae commonly known as Long COVID (LC), a term coined by the patients themselves (1–3).

Initially the syndrome was defined as a wide range of symptoms that continue or develop after two or three months after the acute phase of the infection by the severe acute respiratory syndrome coronavirus 2 (SARS-CoV-2) (4, 5). However, trying to unify the terminology, the definition has been updated to include the duration of the symptoms more precisely (6). Here we will consider long-COVID (LC) when the syndrome lasts from four weeks to three months after COVID-19, and post-COVID (PC), when it lasts from three months to years after COVID-19. LC/PC develops in people of all ages but presents a higher incidence in middle-aged women. It affects several organs and systems with mild, moderate, or severe disease progression. Manifestations of the syndrome mainly involve the circulatory, immune, neurological, and musculoskeletal systems. Frequently reported symptoms are fatigue, brain fog, muscle, chest pain, dyspnea, and heart arrhythmias (7, 8).

Importantly, considering that SARS-CoV-2 is still circulating, we must keep in mind that the risk of developing LC/PC could increase with reinfections (9). Therefore, the development of sequelae after COVID-19 is becoming an increasingly relevant public health problem due to its high incidence and often disabling symptomatology (10).

The clinical characteristics of LC/PC patients and the reasons behind the persistence of the symptoms have been investigated pointing to diverse underlying causes (11), such as infection-related immune dysregulation, autoimmunity, and excessive inflammation (12, 13), incomplete virus clearance (14) and reactivation of latent viruses (15–17), all of which could be interrelated.

Currently, there is an extensive body of literature examining antibody production against SARS-CoV-2 essential proteins, such as the Nucleocapsid and the Spike, their correlation with symptoms and severity during the acute phase of the infection, and in response to vaccination (18, 19). Regarding the Spike, it is a trimeric protein composed of two main domains, the S1 is responsible for the entrance while the S2 is for the virus‐cell membrane fusion. The S1 contains the so-called receptor-binding domain (RBD) (20). Accordingly, numerous studies have focused on determining total immunoglobulin G (IgG) and IgG1 against the S1 and the RBD (21).

However, only a scarce number of studies have investigated the production of IgGs in LC/PC cohorts. One such study reported higher titers of IgG anti-S1 in PC individuals compared to fully recovered infected individuals, though the amounts of Spike and RBD specific IgGs were similar in both groups (22). On the contrary, a second study investigated S1 and RBD Spike IgG antibodies and found decreased antibody titers in PC individuals (23). Therefore, more research is needed to clarify this crucial subject to comprehend the role of the immunological response in the development of PC, which is precisely the focus of the present study. We hypothesized that the antibody response of post-COVID syndrome patients might be altered in comparison to the one of COVID- recovered individuals.

There are four IgG subclasses, enumerated from the highest to the lowest serum concentration as IgG1, IgG2, IgG3, and IgG4. Each subclass has specific characteristics, such as half-life and effector functions, mainly determined by the crystallizable fragment (Fc) of the molecule (24). In this sense, IgG1 and IgG3 play key roles in phagocytosis, cell lysis, and complement activation. IgG2 seems to have a specific affinity for certain polysaccharide antigens such as the ones present in capsulated bacteria (25). Although IgG4 function is not completely understood, it is considered less inflammatory (26). IgG4 is specially produced during type-2 immunity (27) in response to venoms (28) and helminths (29). Therefore, it is considered less protective against most viral infections (26).

Therefore, our major aim was to perform a retrospective study to determine and analyze the anti-Spike IgG subclass response in a PC cohort with respect to a COVID-19 recovered cohort. Besides, to gain further insights into the antibody response, we employed two different strategies by using the full-length Spike and the isolated RBD. Additionally, we determined the anti-Nucleocapsid antibody production for both cohorts, as an indicator of viral response since this SARS-CoV-2 protein was not included in the vaccines used during the vaccination campaigns in Spain. Apart from analyzing the evolution of the symptoms and the most relevant risk factors for developing PC, we present the data regarding antibody production which we analyzed considering the vaccination status and the infection history of individuals in both cohorts.

## 2 Methods

### 2.1 Study design

Patients from the “post-COVID” cohort (PC, n= 104) were diagnosed based on symptoms and medical evaluation (when tests were not yet available), serology or Polymerase Chain Reaction (PCR) tests, and displayed signs and symptoms that develop during or after an infection consistent with COVID-19, present for more than 12 weeks and were not attributable to alternative diagnoses (6). Samples were collected between June 27^th^ and July 8^th^ of 2022. For a subset of PC individuals (n= 36) we were able to collect a previous sample (sub-cohort PC1), between the 9^th^ and 24^th^ March of 2022, which we used for a longitudinal study (PC1 and PC2 sub-cohorts). The post-COVID syndrome cohort included four individuals with three infections. The “COVID” recovered cohort included individuals who recovered from COVID-19 without any sequelae. COVID samples (n= 30) were collected from October 17^th^ to 26^th^ of 2022 to compensate for their later infection. Most of them (77%) were employees at the UCM University. All participants live in the Comunidad de Madrid (Spain). Samples were collected in Alcorcón. In Spain, the vaccination campaign started the 27^th^ of December 2020. 85 patients in the PC cohort were vaccinated (1 dose n= 17, 2 doses n= 47, 3 doses n= 21), and 19 were unvaccinated. The precise type of vaccine and date of vaccination could be registered only for a subset of patients (COVID n= 23 out of 30, PC n= 37 out of 85 vaccinated). Vaccines administered are shown in **Supplementary Table S1**. Relevant data for both cohorts was recorded at the time of sample collection.

To set-up the cut-off for the flow-cytometry assay and as negative controls for the ELISAs, we obtained sera from healthy donors from a biobank, collected before December 2019, referred to as the “pre-pandemic cohort” (“PP”, n= 77).

### 2.2 Jurkat-Spike flow-cytometry method

We used the previously developed assay (30) adapted to detect IgG subclasses. Briefly, the full-length (FL) SARS-CoV-2 Wuhan Spike was expressed in Jurkat T-cells by means of a bicistronic lentiviral construct containing truncated epidermal growth factor receptor (EGFR), as an expression control. Cells were incubated with the serum samples (1/50), and after washing, with subclass-specific labeled secondary antibodies (**Supplementary Figure S1**). EGFR was detected with anti-human EGFR (clone AY13) conjugated with APC or VB 421 (Biolegend). Secondary antibodies were from Southern Biotech: anti-Human Fc IgG-APC (mouse clone 9042-11), anti-Human Fc IgG1-PE (mouse clone HP6001), anti-Human Fc IgG2-PE (mouse clone 31-7-4), anti-Human Fc IgG3-PE (mouse clone HP6050), anti-Human Fc IgG4-PE (mouse clone HP6025), goat anti-human IgA-PE (2050–09). The cellular viability marker used was 7AAD (Beckton Dickinson). Cutt-offs were determined as the mean + 2 S.D. of the PP sera: IgA 11.6, IgG 2.99, IgG1 1.61, IgG2 1.21, IgG3 1.15, IgG4 1.20.

### 2.3 IgG anti-Nucleocapsid and IgG1, IgG2, IgG4 anti-RBD determinations

Commercially available ELISA kits were used as per manufacturer instructions. Nucleocapsid-based ELISA was INgezim COVID-19 DR (Eurofins Ingenasa ref. 50.Cov.K.0/5). RBD ELISA kits were from ACROBiosystems (distributed by Fisher Scientific): Anti-SARS-Cov-2 Antibody Titer Serologic Assay Kit IgG1 (RAS-T014), IgG2 (RAS-T015), and IgG4 (RAS-T017), with Cut-offs: 0.1, 0.1, and 0.2 respectively.

### 2.4 Statistics

The strategy employed includes a general analysis followed by stratified analyses to account for the major factors conditioning the response (31). We used the GraphPad Prism software version 9.0. The χ2 test or the Fisher’s exact test was used for categorical variables. Continuous variables were compared using the non-parametric Mann-Whitney U test, the Wilcoxon test for PC1 and PC2 paired samples, and the Kruskal-Wallis test for multiple comparisons. The *Spearman*’s rank correlation coefficient (*r*_s_) was used to determine the correlation between variables. P values were adjusted as indicated in figure legends and depicted as *P< 0.05; **P< 0.01; ***P< 0.001; and ****P< 0.0001.

## 3 Results

### 3.1 Description of the COVID and post-COVID syndrome cohorts

We studied 104 PC individuals and 30 COVID-19- recovered individuals. 90% are women and 95% Caucasians (only 5 patients are of Hispano-American origin). Similarly, the COVID- recovered cohort included 94% women all of them Caucasians. Median age was similar between both cohorts: 48 with an interquartile range (IQR) of 46–54 for PC, and 51 with an IQR of 44–59 for COVID. Other characteristics are shown in **Supplementary Figure S2**. Students’ *t-*test analyses revealed no significant differences in the mean distribution of weight, height, and body mass index (B.M.I.) (data not shown). First, we used demographic data to calculate the relative risks (RR) for developing PC conferred by the variables (**Supplementary Figure S2A**). Remarkably, we found that individuals under medication displayed the highest significant RR=1.85, 95% confidence interval (C.I.) (1.32- 2.92) (**Supplementary Figure S2A**). **Supplementary Figure S2B** shows additional demographic data that could be obtained regarding race and occupation for some post-COVID patients.

**Figure 1:**
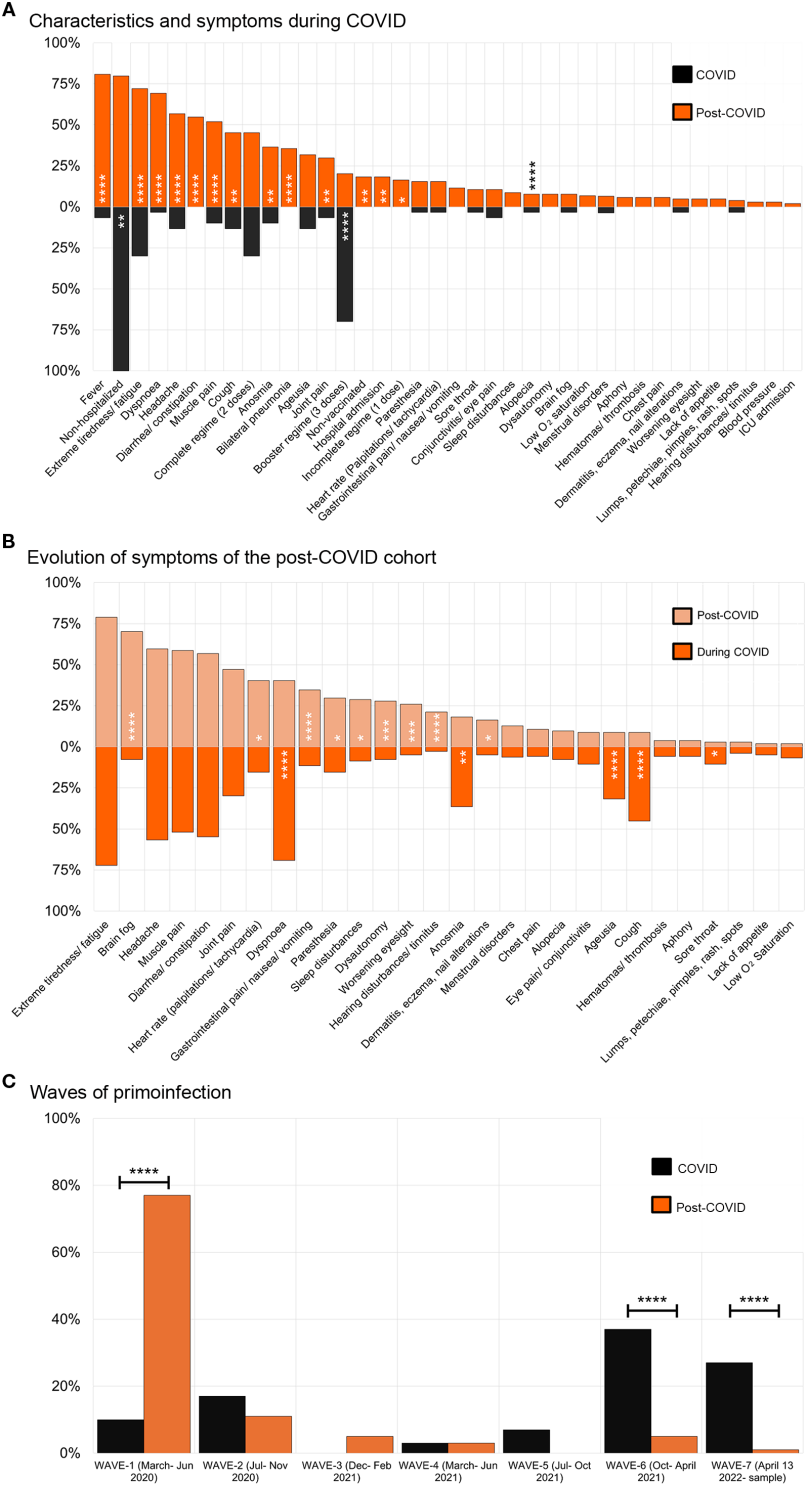
Characteristics and symptoms presented by individuals in the cohorts. **(A)** Symptoms presented during COVID-19 by individuals from the COVID-recovered cohort (black boxes, n= 30) compared to the post-COVID syndrome cohort (orange boxes, n= 104). **(B)** Evolution of symptoms presented by post-COVID syndrome patients during the COVID-19 (orange boxes) compared to the post-COVID syndrome period (light orange boxes), n= 104. The χ2 test was used, or the Fisher’s exact test when n< 5 **(C)** Waves of the primoinfection for individuals from the COVID and post-COVID syndrome cohorts. The different waves in which individuals were infected are shown. Variants of concerns were first detected: original Wuhan strain (waves 1 and 2, part of 3), Alpha, 17 Feb. 2021, Delta, 9 Jun. 2021, Omicron, 13 Jan. 2021. Images were produced with Microsoft Excell and mounted with Adobe Photoshop CS6.

Similarly, we analyzed the symptoms developed during the acute phase of the primoinfection for both cohorts. Interestingly, we found that fever, extreme fatigue, bilateral pneumonia, and infection before vaccination were significantly increased in the PC cohort (**Figure 1A**) and they rendered significant RRs. Especially relevant is the RR of infection before vaccination RR=1.72, 95%, (C.I. 1.33 – 2.38) (**Supplementary Figure S3**). Reinfection with SARS-CoV-2 conferred a RR=1.29, 95%, (C.I. 1.08 – 1.56). The mean ± S.D. time in months from reinfection to sampling was: 6.2 ± 2.7 months for COVID, and 5.4 ± 4.7 for PC samples.

To comprehend the evolution of symptoms in PC individuals we compared their frequency during the acute phase of the primoinfection and the PC period (**Figure 1B**). Extreme fatigue, headache, muscle pain, diarrhea, or constipation were invariable during the period studied. In addition, some symptoms decreased (e.g. dyspnea) while others appeared from “*de novo*”, such as brain fog, heart rate alterations, and hearing disturbances, indicating the dynamic nature of the syndrome.

Next, we analyzed other relevant aspects of the cohorts such as the wave in which the primo-infection took place, according to data from *Comunidad de Madrid* (32) (**Figure 1C**). Even though the cohorts presented differences, we wanted to gather a general perspective of the antibody response (shown in the next point), to proceed afterwards to perform different stratified statistical analysis to include them.

### 3.2 Anti-Nucleocapsid IgG and anti- full length-Spike IgG and subclass antibodies

**Figure 2:**
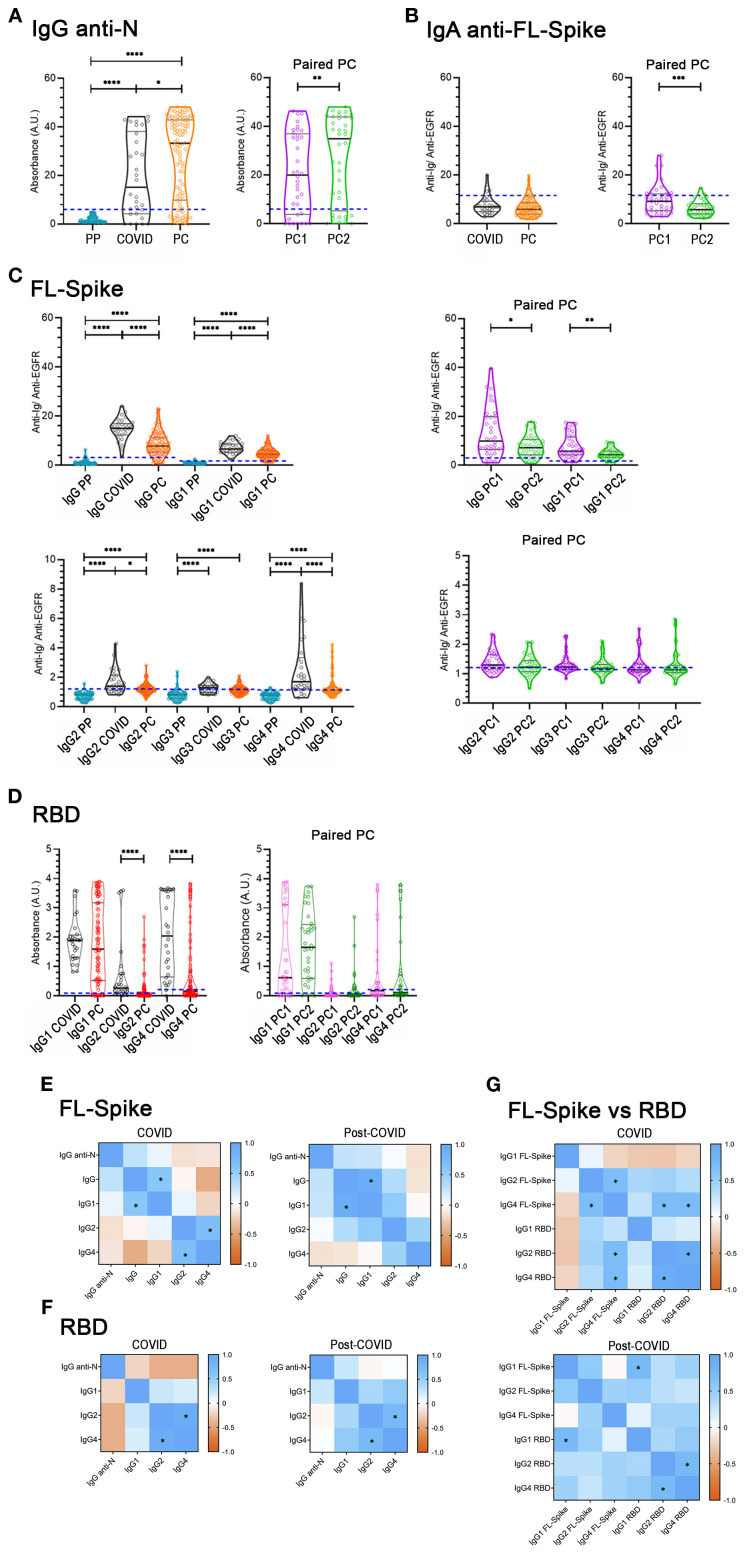
Determination of immunoglobulins in the COVID-recovered cohort, the post-COVID cohort, and in the paired post-COVID sub-cohorts. **(A)** IgG anti-Nucleocapsid (N). **(B)** Anti-full-length (FL) Spike serum IgA antibodies. **(C)** Anti-FL Spike antibodies, total IgG, IgG1, IgG2, IgG3, and IgG4 subclasses. **(D)** Anti-receptor-binding domain (RBD) IgG1, IgG2, and IgG4 subclasses in the COVID (n= 30) and Post-COVID syndrome (n= 104) cohorts (left panels), and in the Post-COVID syndrome paired PC1/PC2 sub-cohorts (right panels, n= 36). Cut-offs are indicated by dashed blue lines. PP: 77 pre-pandemic sera were used to corroborate the specificity of the anti-N IgG ELISA kit, and to set-up the flow-cytometry cut-offs. Determinations were performed once. U-Mann-Whitney test with *post hoc* Bonferroni correction in **(C, D)** was used for COVID/PC comparisons. Wilcoxon test was used for the PC1/PC2 longitudinal study. Data is shown with mean and standard deviation. **(E, F, G)** *Spearman* correlation between variables. * Indicates *r*_s_≥ 0.6 and statistically significant *p*. PC, post-COVID syndrome; vs, versus. Images were produced with GraphPad Prism V9 and mounted with Adobe Photoshop CS6.

First, we quantified IgG antibodies against the Nucleocapsid (N), which was not included in the vaccine regime in Spain. To exclude the possibility of cross-reactions with seasonal coronaviruses, we used 33 prepandemic sera to corroborate the specificity of the ELISA kit, and found that any of the PP samples yield values above the Cut-off= 6 defined by the manufacturer of the ELISA (**Figure 2A**). We found that the mean value of the IgG anti-N sample distribution was increased in the PC cohort compared to the COVID cohort (**Figure 2A**). Similarly, PC1 and PC2 paired samples presented a statistically significant increase (**Figure 2A**). Given the results obtained in the paired analysis (**Figure 2A**), we next compared non-reinfected to samples reinfected during the PC1-PC2 period. We found a statistically significant increase in IgG anti-N for the PC2 reinfected and non-reinfected unpaired comparison (data not shown), which could help to explain the increase initially detected (**Figure 2A**).

Additionally, we determined IgA, total IgG (referred to simply as IgG), and IgG subclasses (IgG1- IgG4) anti-Full length (FL) Spike antibodies (**Figures 2B, C**). Most values regarding serum IgA anti-FL-Spike of COVID and PC cohorts were negative.

Concerning the anti-Full Length (FL) Spike IgG and IgG1- IgG4 subclass sample distributions of both cohorts (**Figure 2C**), except for IgG3, we found that their mean values were significantly decreased in PC compared to the COVID cohort; while in the PC1 and PC2 paired samples, only IgG and IgG1 were diminished over time (**Figure 2 C**, right panels).

Altogether these results (**Figure 2C**) indicate that PC samples have decreased median amounts of anti-FL-Spike IgG and IgG1, IgG2, and IgG4 subclass antibodies and that, for the PC paired sub-cohorts, IgG and IgG1 seem to be more variable over time than IgG2 and IgG4.

### 3.3 Anti-RBD IgG1, IgG2, IgG4 subclass antibodies

The RBD is very immunogenic and relevant as a target for neutralizing antibodies (33). Therefore, we next employed commercially available RBD-based ELISA assays. Nonetheless, considering there were no differences in IgG3 between COVID and PC cohorts (**Figure 2C**), we specifically aimed to study anti-RBD IgG1, IgG2, and IgG4 from this point forward. In contrast to previous results obtained with the FL-Spike (**Figure 2C**), only IgG2 and IgG4 were significantly reduced in PC cohort (**Figure 2D**, left panel). However, the comparison of the PC1 and PC2 paired samples render no differences for IgG1, IgG2 and IgG4 anti RBD (**Figure 2D**, right panel).

Finally, we examined the level of correlation between the different immunoglobulins determined for the Nucleocapsid, the FL-Spike and the RBD, by calculating the *Spearman*’s rank correlation coefficient (*r*_s_) (**Figures 2E–G**, and **Supplementary Table S2**). We considered as relevant correlations those with an *r*_s_ ≥ 0.6 and a statistically significant *p*. After applying the criteria, we detected a correlation for both cohorts between FL-Spike IgG and IgG1 (COVID *r*_s_= 0.6; p< 0.001; PC *r*_s_= 0.9; p< 0.001) and between RBD IgG2 and IgG4 (COVID *r*_s_= 0.9; p< 0.0001; PC *r*_s_= 0.7; p< 0.0001). However, FL-Spike IgG2 and IgG4 only correlated in samples from the COVID cohort (COVID *r*_s_= 0.7; p< 0.0001) and not in the ones from the PC cohort (PC *r*_s_= 0.5; p< 0.0001).

The data might indicate differences in the antibody response pattern between the two cohorts that prompted us to perform further analysis, this time comparing antibodies against the FL-Spike and the RBD, despite the assays´ differing sensitivities. The results shown in **Figure 2G** corroborated the different response patterns. Interestingly, a correlation between IgG1 anti-FL-Spike and IgG1 anti-RBD was exclusively detected in samples from the PC cohort.

### 3.4 Analysis considering the vaccination regime

**Figure 3:**
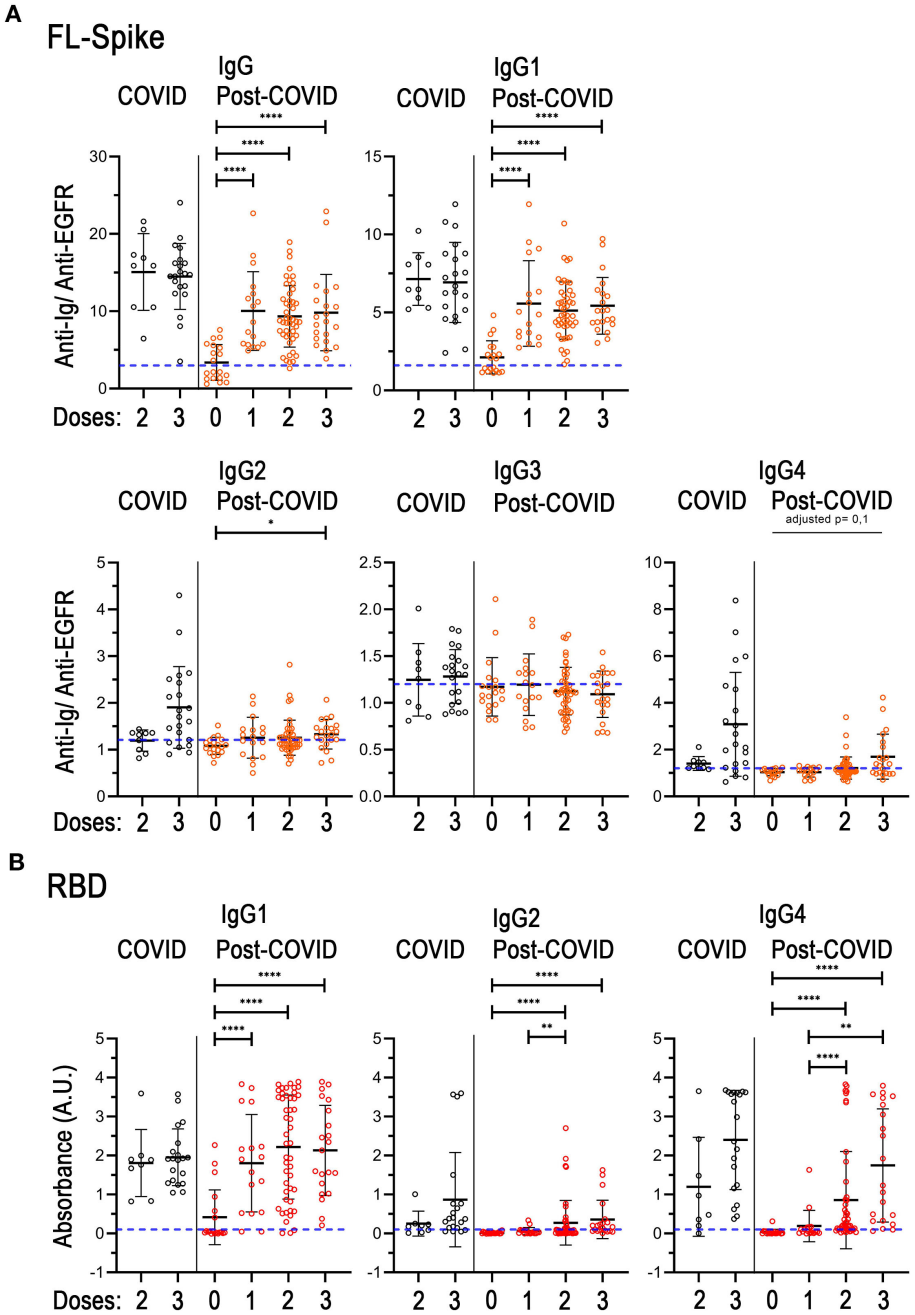
Analysis of immunoglobulins considering the individual vaccination regimes in the COVID-recovered and post-COVID cohorts. **(A)** Total IgG, and IgG1, IgG2, IgG3, IgG4 subclasses against full-length (FL) Spike. **(B)** IgG1, IgG2 and IgG4 subclasses against the receptor-binding domain (RBD). COVID n= 9, (8 for B) 2 doses, n= 21 (20 for B) 3 doses. Post-COVID n= 19 (0 doses), n= 17 (1 dose), n= 47 (2 doses), n= 21 (3 doses). The U-Mann-Whitney test with *post hoc* Bonferroni correction was used for post-COVID comparisons. Data is shown with mean and standard deviation. Abbreviations: doses, vaccine doses. Images were produced with GraphPad Prism V9 and mounted with Adobe Photoshop CS6.

In healthy individuals, the change in vaccination status from unvaccinated and one vaccine dose to two mRNA vaccine doses progressively increases the titter of IgG1 anti-Spike antibodies produced, while the change from two to three fundamentally favors a switch towards the production of IgG2 and IgG4 (34). Regarding the anti-RBD response, mainly the change from the second to the third vaccine dose promotes an increase in IgG4 (34). Consequently, we proceeded to subdivide the COVID and PC cohorts in different subsets according to the number of vaccine doses received by the different individuals, and re-analyzed both the anti-FL-Spike and the anti-RBD IgG determinations (**Figure 3**).

Unfortunately, the COVID cohort did not include either unvaccinated individuals or individuals with only one vaccine dose, therefore we could not detect the expected augmented production of IgG1 after the first vaccination dose. Hence, the mean of IgG and IgG1 sample distributions were noticeable but similar between the two and three dose subsets, as expected (**Figure 3A**). Additionally, although the differences were not statistically different, in the third-dose vaccine group there were perceptible increases in the amounts of IgG2 and IgG4 against both FL-Spike and RBD, in agreement with previous reports (34). Regarding IgG3 anti-FL-Spike, we found that the amounts of this immunoglobulin remain unchanged in all subsets from both cohorts (**Figure 3A**).

Remarkably, when we compared the unvaccinated to the one-dose group in the PC cohort, a significant increase in the mean of the sample distribution of IgG and IgG1 anti FL-Spike and anti-RBD was observed (**Figure 3**). In addition, the mean of the one-dose group was comparable to the one displayed by individuals with two or three vaccine doses (**Figure 3**). On the other hand, IgG2 and IgG4 anti-RBD were increased in individuals with the second dose and remained at similar levels in individuals with the third vaccine dose (**Figure 3B**).

Next, we represented the antibody values against FL-Spike and RBD combined for each sample from the second and third vaccine subsets (**Supplementary Figure S4**). We could appreciate that IgG1 depiction was alike for both COVID and PC cohorts showing that high anti-FL corresponded with high anti-RBD (parallel lines for most of the sera) but with steeper slopes in the first case. Additionally, because the subsets analyzed (**Figures 3**, **Supplementary Figure S4**) presented different percentages of reinfected individuals (shown in **Supplementary Table S3**), we performed the same analysis but this time we included only non-reinfected patients, which showed comparable results (**Supplementary Figure S5**).

Altogether the results in **Figures 3** and **S5** might indicate the existence of differences on IgG4 subclass switch between COVID and PC individuals after the second and third vaccine dose that we further investigated.

**Figure 4:**
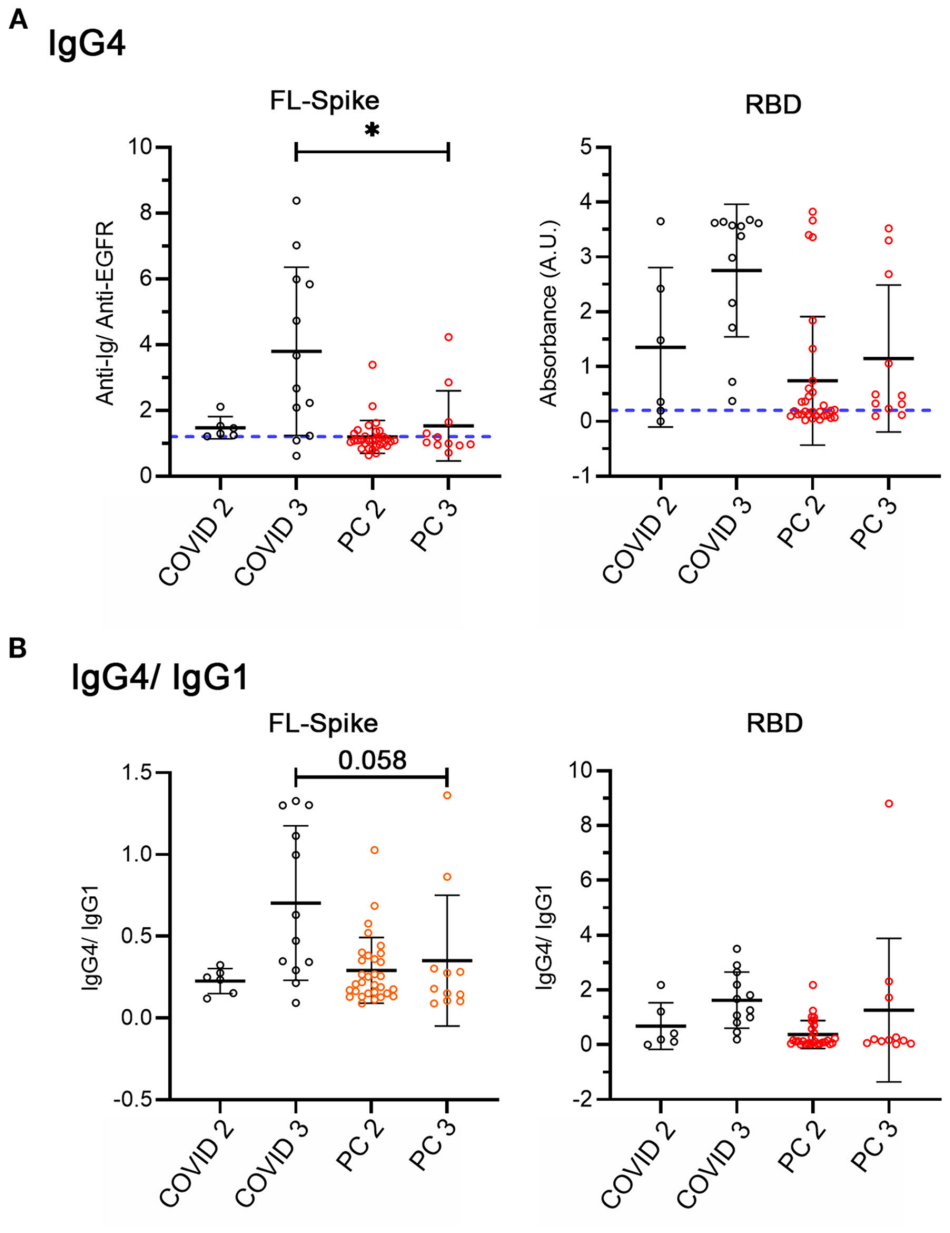
Analysis of IgG4 and IgG4/IgG1 ratio considering non-reinfected COVID-recovered and post-COVID individuals with two and three mRNA vaccine doses. **(A)** IgG4 against full-length (FL) Spike and the receptor-binding domain (RBD). **(B)** IgG4/IgG1. COVID n= 6 (2 doses), n= 12 (3 doses), PC n= 31 (2 doses), n= 11 (3 doses). All conditions were compared with the Kruskal-Wallis test with *post hoc* Dunn test. Data is shown with mean and standard deviation. FL, full length, PC, post-COVID syndrome; RBD, receptor-binding domain. Images were produced with GraphPad Prism V9.

Consequently, we compared the values of IgG4 for two and three vaccine doses subsets and calculated the IgG4/IgG1 ratio because it is frequently used in medical practice, as it normalizes the values for each serum. Furthermore, it has been used to study the antibody response in COVID patients (35). We performed the analysis (**Figure 4**) excluding individuals that received vector-based vaccines because the switch has been specifically studied after mRNA vaccination (34), and for homogeneity (**Supplementary Table S3**), excluding reinfected individuals.

Even though the number of samples was small, we found decreased IgG4 anti-Spike in post-COVID syndrome patients (**Figure 4A**) but not clear indications of differences in the IgG4/IgG1 switch (**Figure 4B**). Therefore, to end, we analyzed the effect of reinfections directly.

### 3.5 Effect of vaccination and a recent infection

Comprehensibly, SARS-CoV-2 infections condition the antibody response as previously studied for numerous COVID cohorts (18). Accordingly, we subdivide the second and third vaccine dose subsets to consider a recent infection. This implies individuals who suffered a post-Omicron reinfection. We chose six months before the collection of the samples because this time is a relevant threshold for the waning of the antibody response in COVID cohorts (36, 37), and to fix this parameter. Additionally, for homogeneity we selected only those samples from individuals who had the primoinfection in 2020, when the original Wuhan strain was circulating, and before the start of the vaccination campaign.

**Figure 5:**
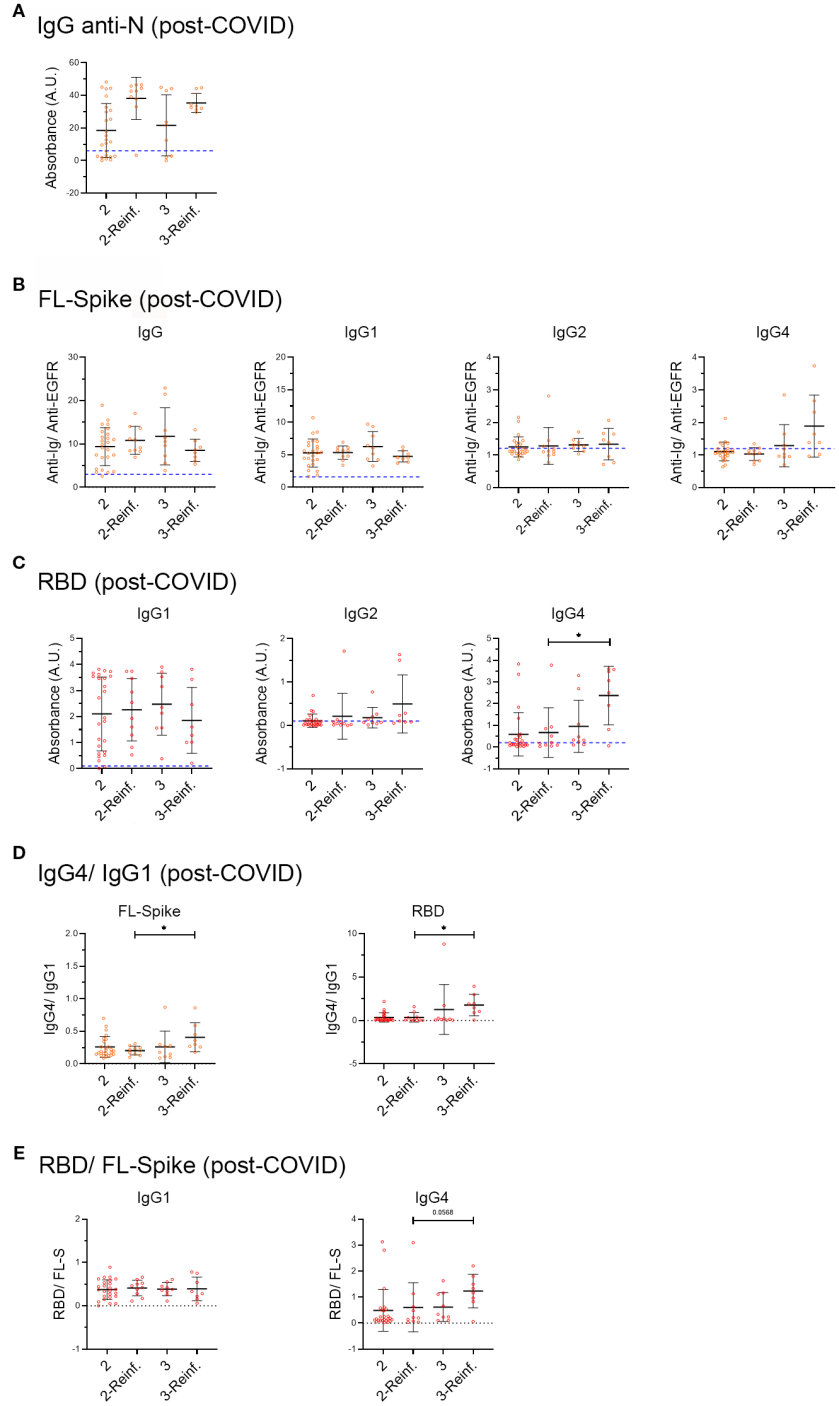
Analysis of samples from post-COVID syndrome patients, infected before vaccination, who afterwards received two or three vaccine doses, and with or without a recent six-month reinfection. **(A)** IgG anti-Nucleocapsid (N), **(B)** anti-full-length (FL) Spike IgG, IgG1, IgG2, IgG3 and IgG4 subclasses and **(C)** anti-receptor-binding domain (RBD) IgG1, IgG2 and IgG4 subclasses in the COVID- recovered and Post-COVID syndrome cohorts. **(D)** IgG4/IgG ratio. **(E)** RBD/FL-Spike ratio. PC cohort individuals infected in 2020 before vaccination were subdivided into two and three vaccine dose subsets, as well as a recent SARS-CoV-2 reinfection that occurred within the 6 months preceding sample collection. We included individuals for which the exact date of vaccination was known and those who were infected before the start of the vaccination campaign in Spain (27 of December 2020). Post-COVID: two doses n= 25, reinfected n= 9, three doses n= 9, reinfected n= 8. Analyses were performed using the Kruskal-Wallis test with *post hoc* Dunn test. Data is shown with mean and standard deviation. reinf, reinfection. Images were produced with GraphPad Prism V9 and Adobe Photoshop CS6.

Unfortunately, due to the reduced number of COVID samples we could include, we decided to compare only the PC subsets with 2 and 3 vaccine doses, reinfected and no reinfected (**Figure 5**). **Supplementary Table S4** shows relevant characteristics of the samples analyzed in **Figure 5**. We noticed a different percentage of PC patients that had pneumonia during the primoinfection in the reinfected (22%) and non-reinfected subsets (56%). However, it did not yield a statistically significant different level of the immunoglobulins analyzed (data not shown).

Although it did not reach statistical significance, we observed that while there was an increase in the median of IgG anti-N in reinfected individuals, as previously reported for COVID-recovered cohorts (18), the antibody response against the Spike and the RBD did not change, with the exception of anti-RBD IgG2 and IgG4 that increased (**Figure 5**).

Next, we calculated the IgG4/IgG1 ratio (**Figure 5D**), and as extra control, the RBD/FL-Spike ratio (**Figure 5E**). The results seem to indicate that an increased in anti-RBD IgG4 occurred in recently reinfected PC samples with 3 vaccines doses.

Altogether, the results in **Figure 5** may indicate that PC individuals infected before vaccination, who later received 2 or 3 vaccine doses, do not sufficiently increase IgG and IgG1 anti-FL Spike and anti-RBD after a reinfection. At the same time, they switch to produce IgG4, mainly focused on the RBD, which is especially evident in individuals with 3 vaccine doses and a recent reinfection (**Figure 5**).

## 4 Discussion

Although many aspects of the immunological response in post-COVID syndrome (PC) patients have been investigated (12, 38), the antibody response remains inadequately understood. Here, we specifically addressed such matter by determining the production of antibodies against the Nucleocapsid (N), the full-length (FL) Spike and the RBD, with a special focus on IgG subclasses.

In general, and in agreement with previous reports, the post-COVID cohort studied here presents the expected risk factors, such as fever, fatigue, and bilateral pneumonia (39). Notably, infection before vaccination rendered a significant risk, RR=1.7, 95% CI (1.3 – 2.4). This result confirms that vaccination contributes to the prevention of post-COVID syndrome, as previously reported (40). However, a limitation is that our analysis did not include relevant health habits, such as smoking, which has been previously shown to increase the risk for developing post-COVID syndrome (41).

On the other side, the evolution of symptoms of the post-COVID cohort followed a trend comparable to the one previously described, with the improvement of some of them, especially respiratory ones such as “cough”, but worsening or appearance of others, e.g. “brain fog” (7).

In the initial general analysis, we detected that the levels of anti-FL-Spike IgG and IgG subclasses in the PC cohort were lower than in the COVID cohort, while the anti- N IgG median value was increased (**Figure 2**). Regarding the antibody response against the RBD, it was interesting that IgG1 median values were similar between both cohorts, while IgG2 and IgG4 were diminished in the PC cohort (**Figure 2**). In this respect, a limitation of our study is that we could not determine IgG3 anti-RBD (**Figure 2D**) to corroborate the lack of difference found for the anti-FL-Spike IgG3 (**Figure 2C**).

Regarding the PC1-PC2 sub-cohort longitudinal analysis (right panels of **Figures 2A–D**), it is remarkable that the IgG anti-N augmented, IgG and IgG1 anti-FL Spike decreased, while the IgG1 anti-RBD remained unchanged, as in the general analysis (**Figures 2A–D** left panels). It indicates that post-COVID patients produce increased levels of IgG against the Nucleocapsid but low levels of IgG anti-FL-Spike, while the IgG1 response to the SARS-CoV-2 RBD is conserved.

We might speculate that the impaired response of PC patients to the FL-Spike can be explained by “immunological imprinting” resulting from previous infections with seasonal coronaviruses, as proposed in a very recent study (42), published when this manuscript was under review. Interestingly, the study shows that PC patients present higher levels than healthy controls of IgG anti-Spike from OC43, HKU1, NL63, and 229E seasonal coronaviruses, which possess Spike proteins highly homologous to the SARS‐CoV‐2 Spike protein (42). Importantly, using an in-house ELISA kit, the researchers detected decreased IgG anti- SARS-CoV-2 Spike in PC patients compared to healthy controls, which agrees with in our results using the cytometry method. Even more, increased IgG anti-Nucleocapsid was also reported in the PC cohort (42).

In accordance with the “original sin immunology theory”, the authors suggested that the antibodies would be less effective towards the second antigen, in this context, the Spike from SARS-CoV-2. This theory proposes that when B cells are re‐exposed to an antigen (Spike from SARS-CoV-2) that is very similar to an original antigen to which B cells were first exposed to (Spike from seasonal coronaviruses), the response that predominates is the one derived from memory B cells specific for the first antigenic encounter, and therefore, an antibody response would be mounted predominantly to the original antigen, instead of the second antigen (42).

In our study, we cannot exclude that the differences found in the general analyses performed (**Figure 2**) can be partially attributable to the different pattern of infection and vaccination of both cohorts (**Figure 1** and **Supplementary Table S1**), or to possible confounders we have not detected. For example, the COVID-recovered cohort includes mainly individuals that were infected after vaccination (**Supplementary Figure S3**), while for the PC cohort the situation is the opposite. However, we must consider that the results are confirmed by the stratified analysis, for example the level of anti-FL-Spike remains much lower in PC samples than in COVID-recovered samples even when they are split by the number of vaccine doses received (**Figure 3**; **Supplementary Figure S5**), as explained next.

Trying to counteract the cohort differences, we proceed to analyze the vaccination response of both cohorts considering the number of vaccine doses (**Figure 3**, S4) and without including reinfected individuals (**Supplementary Figure S5**). We were able to notice that the PC cohort increased significantly the titers of IgG and IgG1 after the first vaccine dose. However, except for RBD IgG1, PC patients seem to respond fainter to the vaccine than COVID recovered individuals (**Figures 3**, **S5**).

Additionally, it has been reported that the IgG anti-Spike response after the third vaccine dose of individuals with naïve (not infected before vaccination), and with hybrid immunity (vaccinated and infected) (43), as well as the response to different types of vaccines of previously infected individuals, tends to be similar (44). In other words, if the antibody responses of COVID individuals and PC patients after several exposures, such as the third vaccine dose plus a reinfection, were similar, we would have detected an increase in the levels of IgG anti-Spike in the latter group, which was not the case.

Next, we evaluated the IgG class switch in non-reinfected individuals who received two and three mRNA vaccine doses (**Figure 4**). We found no differences in the normalized IgG4/IgG1 ratio, although the tendency was that proportionally, there were more COVID samples that presented high levels of IgG4. This finding was corroborated in **Supplementary Figure S5**, when we analyzed samples considering vaccine doses in non-reinfected individuals. We detected that after the third vaccine dose the COVID recovered cohort had significantly higher anti-RBD IgG4 levels than PC patients (**Supplementary Figure S5B**).

Notably, the analysis of PC samples from vaccinated individuals with two and three vaccine doses, who were infected before vaccination, in 2020 by the Wuhan strain, subdivided in non-reinfected or reinfected in the post-Omicron period, shows that anti-N IgG antibodies were readily detectable in PC reinfected samples (**Figure 5**). In contrast, anti-FL-Spike IgG, and IgG1 and anti-RBD IgG1 were unchanged in them, while we were able to detect the anti-RBD IgG4 switch. Due to the small number of COVID-recovered participants, we could not compare the PC to the COVID samples in **Figure 5** (limitation). However, other studies have reported that COVID-recovered individuals with two or three vaccine doses respond to reinfection with a detectable increase in the production of IgG anti-Spike (43).

Furthermore, future studies should corroborate whether the increased RBD IgG4/IgG1 ratio, (**Figure 5**) detected in PC patients who were reinfected after the third vaccine dose, also occurs in COVID-recovered individuals. In other words, whether this finding is a specific characteristic of the antibody response of post-COVID patients, or, on the contrary, represents a normal response to several RBD exposures, from both the vaccine and SARS-CoV-2 reinfections. In our opinion, this is a noteworthy discovery that might indicate the suitability of investigating the use of vaccines not solely based on the S1 Spike domain, and perhaps different types of vaccines (45).

Strikingly, we could only detect a correlation between IgG1 anti-FL-spike and IgG1 anti-RBD in PC individuals (**Figure 2G**). Therefore, PC patients seem to have an IgG1 antibody response that is more focalized on the RBD. This might perhaps contribute to the immunopathology of the PC because IgG antibodies specifically targeting the RBD can possess pro-thrombotic properties due to cross-reactions with platelet factor-4 (46), among other reasons.

The clear response to the Nucleocapsid detected would agree with the increasingly supported hypothesis of the persistence of the SARS-CoV-2 in at least some PC-afflicted individuals. Is it possible that those patients present Nucleocapsid reservoirs? In fact, SARS-CoV-2 antigens can be detected in the blood up to 14 months after infection (47) and the Nucleocapsid was found in the muscles from post-mortem biopsies (48). We can speculate that PC individuals presenting an elevated anti-Nucleocapsid IgG response would benefit from antiviral-based pharmacological interventions (49).

To end, our results describing a lower magnitude of the antibody response of PC individuals against the Spike would agree with a successful intervention based on a combination of two SARS-CoV-2 neutralizing monoclonal antibodies (50), and with the fact that intravenous or subcutaneous immunoglobulin treatments have been recently reported to alleviate PC symptoms (51, 52).

Undoubtedly, more research would be required, especially clinical trials addressing the various open questions that our study has conveyed.

## Data Availability

The raw data supporting the conclusions of this article will be made available by the authors, without undue reservation.
