# Supplementary material for: Post-COVID syndrome patients show reduced anti-Spike antibodies compared to COVID-recovered controls, but enhanced IgG4/IgG1 switch after the third vaccine dose
Source: Front Immunol. 2025 Oct 2;16:1670324. doi: 10.3389/fimmu.2025.1670324 (PMC12528128; doi:10.3389/fimmu.2025.1670324)
Supplement: Supplementary file 1 [file DataSheet1.pdf]

Supplementary Material

1 Supplementary Figures and Tables

1.1 Supplementary Figures

Supplementary Figure 1

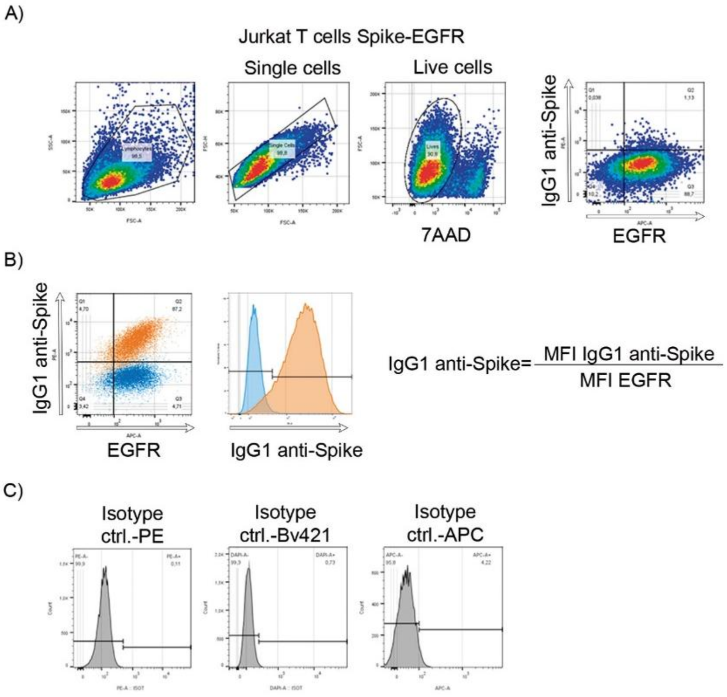

Supplementary Figure 1. Flow cytometry strategy. (A) Gating strategy. Jurkat T-cells expressing the full-length Spike and truncated epidermal growth factor receptor (EGFR) were selected as single live cells using the 7AAD marker. A pool of 77 pre-pandemic sera is used to set the negative threshold (markers) in each assay. **(B)** Example of a dot-plot and a histogram for the pool of pre-pandemic sera (in blue) and an IgG1 anti-Spike positive serum (in orange). The ratio is calculated using the mean fluorescence intensity (MFI) from the specific signal (anti-immunoglobulin) divided by the MFI from anti-EGFR. **(C)** Isotype controls (ctrl.) are shown. Images were produced with Flow Job and mounted with Adobe Photoshop CS6.

Supplementary Figure 2

A) Demographic data

Categories	Variables	Characteristics of the cohort	RR (95% CI)	P value		n (%) COVID	n (%) Post-COVID
Demographic characteristics	Male		0.99 (0.64 - 1.23)	>0.9999	ns	3 (10.0%)	10 (9.6%)
	Female		1.01 (0.81 - 1.57)	>0.9999	ns	27 (90.0%)	94 (90.3%)
	Low BMI (< 18.5)		1.30 (0.73 - 1.43)	0.5868	ns	0 (0.0%)	5 (4.8%)
	Standard BMI (18.5- 25)		0.88 (0.74 - 1.07)	0.2027	ns	22 (73.3%)	62 (59.6%)
	Overweight BMI (25- 30)		0.94 (0.70 - 1.15)	0.6126	ns	7 (23.3%)	20 (19.2%)
	Obesity BMI (> 30)		1.26 (0.98 - 1.45)	0.0741	ns	1 (3.3%)	17 (16.4%)
Morbidities	Allergies/ intolerances		0.86 (0.59 - 1.09)	0.268	ns	7 (23.3%)	15 (14.4%)
	Asthma/ COPD/ other respiratory disorders		1.06 (0.67 - 1.29)	>0.9999	ns	2 (6.7%)	9 (8.7%)
	Autoimmunity		1.10 (0.75 - 1.32)	0.732	ns	2 (6.7%)	11 (10.6%)
	Cancer		0.64 (0.19 - 1.10)	0.2165	ns	2 (6.7%)	2 (1.9%)
	Diabetes		1.29 (0.27 - 2.83)	>0.9999	ns	0 (0.0%)	1 (0.9%)
	Hypertension		1.20 (0.84 - 1.39)	0.2982	ns	1 (3.3%)	11 (10.6%)
	Hyperthyroidism		1.30 (0.66 - 2.86)	0.5745	ns	0 (0.0%)	4 (3.9%)
	Hypothyroidism		1.20 (0.93 - 1.40)	0.1595	ns	2 (6.7%)	19 (18.3%)
	Other infections previous week		0.96 (0.52 - 1.24)	>0.9999	ns	6 (6.7%)	6 (5.8)
	Recent GI alteration		1.28 (1.07 - 1.54)	0.0108	*	6 (20.0%)	49 (47.1%)
	**Medication**		**1.85 (1.32 - 2.92)**	<0.0001	****	15 (50.0%)	78 (77.2%)

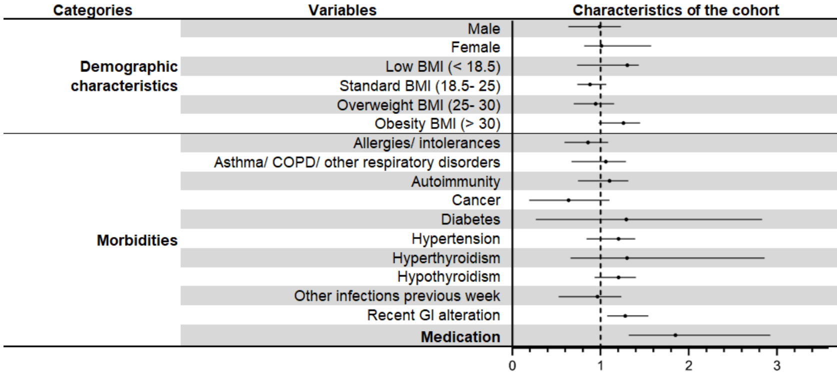

B) Other demographic data

	Post-COVID cohort race and profession (n= 18)	
	**Race**	**Profession**
**1**	Caucasian	Administrative work in reproductive clinic
**2**	Caucasian	Retail sector (online)
**3**	Hispano-American	International business
**4**	Caucasian	Social services
**5**	Caucasian	IT sector
**6**	Caucasian	Hotel manager and sports instructor
**7**	Caucasian	Human Resources technician
**8**	Caucasian	University professor
**9**	Caucasian	Economist (international company)
**10**	Caucasian	IT sector
**11**	Caucasian	Gardener
**12**	Caucasian	Photo interpreter operator (only summer months)
**13**	Caucasian	Consultant engineer
**14**	Caucasian	Web designer student
**15**	Caucasian	Nurse
**16**	Caucasian	Supermarket cashier
**17**	Caucasian	City hall administration technician
**18**	Caucasian	Nephrologist

Supplementary Figure 2. (**A) Demographic data and morbidities present in individuals before the pandemic as risk factors for developing post-COVID syndrome**. Relative risks (RR) conferred by the variables are depicted. In bold RR> 1.5 with significant χ^2 tests. Abbreviations: BMI, body mass index, CI, confidence interval, COPD, chronic obstructive pulmonary disease, GI, gastrointestinal. Images were produced with Microsoft Excell. **(B)** Other demographic data for post-COVID cohort. Information for race and profession for 18 post-COVID individuals could be collected during the manuscript review process.

Supplementary Figure 3

Variable	COVID	RR (95% CI)	P value		n (%) COVID	n (%) Post-COVID
**Hospitalization**	Non-hospitalized	0.74 (0.65 - 0.88)	0.0039	**	30 (100.0%)	83 (79.8%)
	ICU admission	1.35 (1.12 - 2.94)	0.0071	**	0 (0.00%)	2 (1.9%)
	Hospital admission	1.29 (0.44 - 2.84)	>0.9999	ns	0 (0.0%)	19 (18.3%)
**Vaccination regime**	Non-vaccinated	1.35 (1.12 - 2.94)	0.0071	**	0 (0.0%)	19 (18.3%)
	Incomplete regime (1 dose)	1.35 (1.09 - 2.85)	0.0129	*	0 (0.0%)	17 (16.4%)
	Complete regime (2 doses)	1.15 (0.95 - 1.38)	0.1485	ns	9 (30.0%)	47 (45.2%)
	Booster regime (3 doses)	0.55 (0.39 - 0.72)	<0.0001	****	21 (70.0%)	21 (20.2%)
**Others**	Reinfected by SARS-CoV-2	1.29 (1.08 - 1.56)	0.0064	**	6 (20.0%)	50 (48.1%)
	**1st infection before vaccination**	**1,72 (1.33 - 2,38)**	<0.0001	****	9 (30.0%)	78 (77.2%)
**Cardiovasculatory system**	Blood pressure	1.30 (0.57 - 11.39)	>0.9999	ns	0 (0.0%)	3 (2.9%)
	Chest pain	1.31 (0.79 - 2.88)	0.337	ns	0 (0.0%)	6 (5.8%)
	Heart rate (palpitations/ tachycardia)	1.25 (0.96 - 1.44)	0.1183	ns	1 (3.3%)	16 (15.4%)
	Hematoma/ thrombosis	1.29 (0.44 - 2.84)	>0.9999	ns	0 (0.0%)	6 (5.8%)
**Digestive system**	Diarrhea/ constipation	1.64 (1.57 - 1.82)	<0.0001	****	0 (0.0%)	57 (54.8%)
	GI pain/ nausea/ vomiting	1.33 (1.00 - 2.94)	0.0676	ns	0 (0.0%)	12 (11.5%)
	Lack of appetite	1.30 (0.73 - 1.43)	0.5868	ns	0 (0.0%)	5 (4.8%)
**Endocrine system**	Menstrual disorders	1.11 (0.63 - 1.34)	0.337	ns	1 (3.7%)	6 (6.4%)
**Eyesight**	Conjunctivitis/ eye pain	1.10 (0.75 - 1.32)	0.732	ns	2 (6.7%)	11 (10.6%)
	Worsening eyesight	1.30 (0.73 - 1.43)	0.5868	ns	0 (0.0%)	5 (4.8%)
**Immune system**	**Fever**	**2.34 (1.75 - 3.39)**	<0.0001	****	2 (6.7%)	84 (80.8%)
**Musculoskeletal system**	**Extreme tiredness/ fatigue**	**1.54 (1.24 - 2.03)**	<0.0001	****	9 (30.0%)	75 (72.1%)
	Joint pain	1.30 (1.09 - 1.52)	0.0082	**	2 (6.7%)	31 (29.8%)
	Muscle pain	1.46 (1.24 - 1.77)	<0.0001	****	3 (10.0%)	54 (51.9%)
**Nervous system and mental health**	Ageusia	1.22 (1.00 - 1.44)	0.0629	ns	4 (13.3%)	33 (31.7%)
	Anosmia	1.31 (1.10 - 1.54)	0.0062	**	3 (10.0%)	38 (36.5%)
	Brain fog	1.16 (0.73 - 1.36)	0.6832	ns	1 (3.3%)	8 (7.7%)
	Dysautonomy	1.31 (0.88 - 2.89)	0.1982	ns	0 (0.0%)	8 (7.7%)
	Headache	1.48 (1.24 - 1.82)	<0.0001	****	4 (13.3%)	59 (56.7%)
	Hearing disturbances/ tinnitus	1.30 (0.57 - 11.39)	>0.9999	ns	0 (0.0%)	3 (2.9%)
	Paresthesia	1.25 (0.96 - 1.44)	0.1183	ns	1 (3.3%)	16 (15.4%)
	Sleep disturbances	1.32 (0.92 - 8.52)	0.2071	ns	0 (0.0%)	9 (8.7%)
**Respiratory system**	Aphony	1.31 (0.79 - 2.88)	0.337	ns	0 (0.0%)	6 (5.8%)
	**Bilateral pneumonia**	**1.88 (1.53 - 2.46)**	<0.0001	****	0 (0.0%)	37 (35.6%)
	Cough	1.45 (1.45 - 1.45)	0.0013	**	4 (13.3%)	47 (45.2%)
	Dyspnoea	1.34 (1.14 - 1.61)	<0.0001	****	1 (3.3%)	72 (69.2%)
	Low O_2 saturation	1.31 (0.84 - 5.77)	0.3484	ns	0 (0.0%)	7 (6.7%)
	Sore throat	1.20 (0.84 - 1.39)	0.2982	ns	1 (3.3%)	11 (10.6%)
**Skin**	Alopecia	1.16 (0.73 - 1.36)	<0.0001	****	1 (3.3%)	8 (7.7%)
	Dermatitis, eczema, nail alterations	1.08 (0.56 - 1.32)	>0.9999	ns	1 (3.3%)	5 (4.8%)
	Lumps, petechiae, rash	1.16 (0.73 - 1.36)	>0.9999	ns	1 (3.3%)	4 (3.9%)

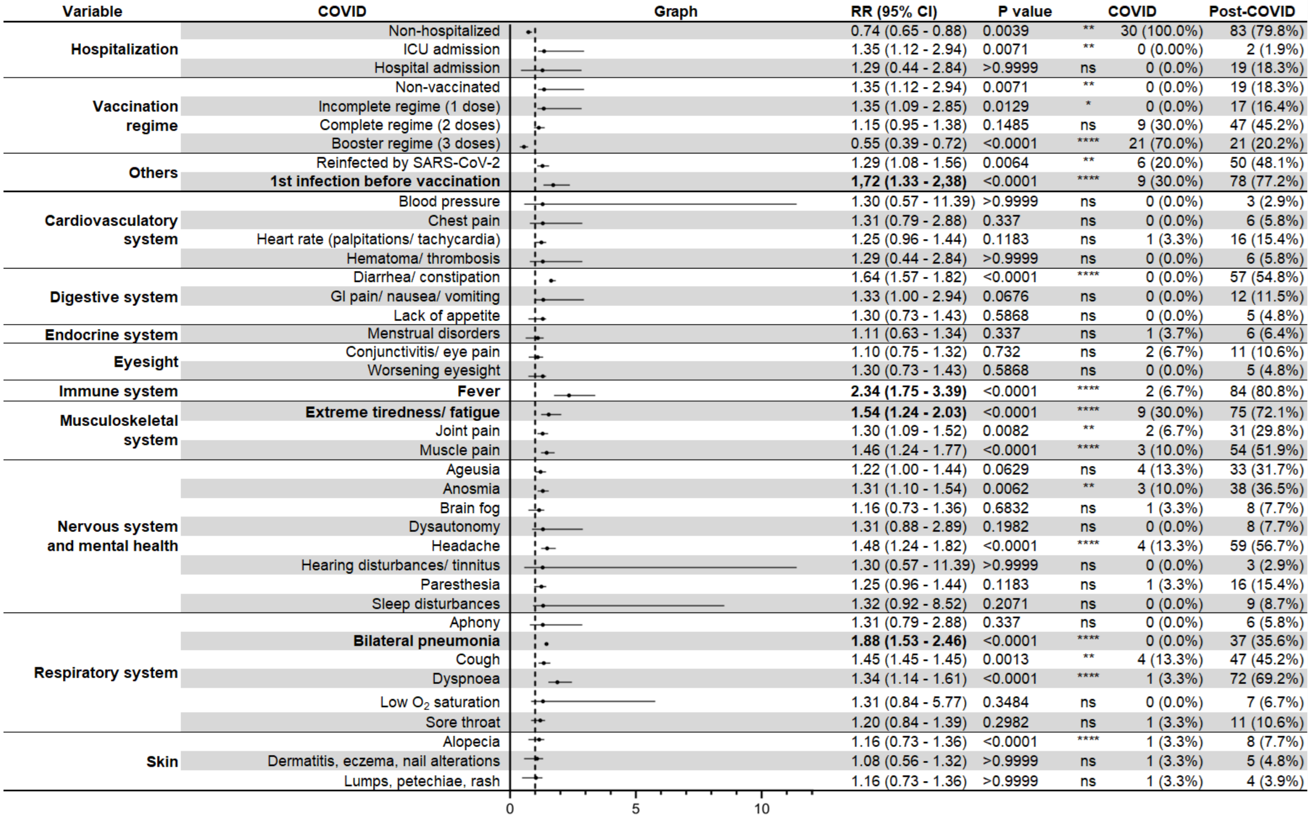

Supplementary Figure 3. **Variables during COVID-19 as risk factors for developing post-COVID syndrome**. Relative risks (RR) conferred by COVID-19 severity measured by hospitalization and ICU admission, vaccination regime, comorbidities, and other relevant factors registered at the moment of the collection of samples. In bold RR> 1.5 with significant χ^2 tests. Abbreviations: BMI, body mass index, COPD, chronic obstructive pulmonary disease, GI, gastrointestinal, ICU, intensive care unit. Images were produced with Microsoft Excell.

Supplementary Figure 4

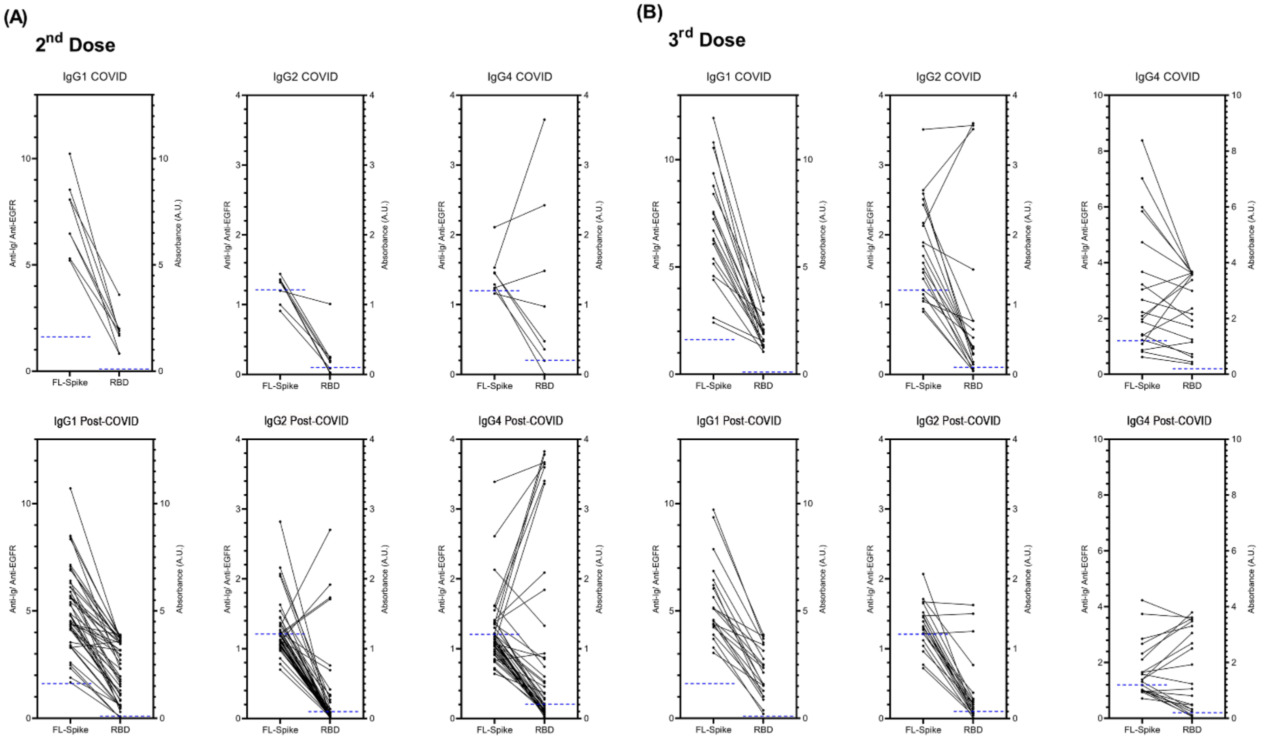

Supplementary Figure 4. Combined depiction of FL-Spike and RBD IgG1, IgG2 and IgG4 subclass values. Representation of IgG1, IgG2, and IgG4 against the full-length (FL)-Spike and the receptor binding domain (RBD) for each individual sample, after subdividing them into two and three vaccine dose subsets for the COVID **(A)** and post-COVID cohorts **(B)**. COVID n= 8 (2 doses), n= 20 (3 doses), PC n= 47 (2 doses), n= 21 (3 doses). Abbreviations: FL, full length. Images were produced with GraphPad Prism V9.

Supplementary Figure 5

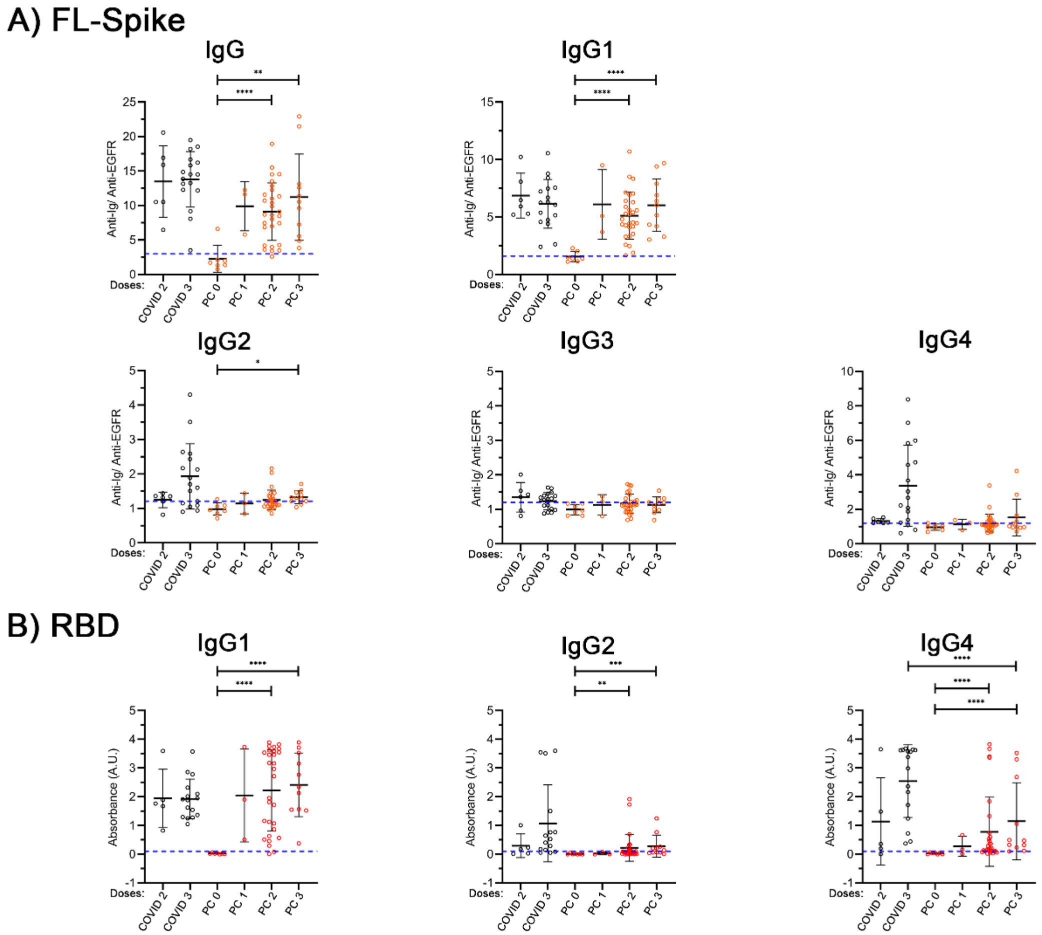

Supplementary Figure 5. Analysis of vaccination in non-reinfected individuals from the COVID-recovered and post-COVID cohorts. (A) Total IgG and IgG1, IgG2, IgG3, and IgG4 subclasses against the full-length (FL) Spike. **(B)** IgG1, IgG2, and IgG4 subclass against the receptor-binding domain (RBD). COVID n= 6 (2 doses), n= 17 (3 doses), PC n= 7 (0 doses), n= 3 (1 dose), n= 29 (2 doses), n= 11 (3 doses). The Mann-Whitney U-test with post hoc Bonferroni correction was used. Data is shown with median and standard deviation. COVID samples are shown in black, PC samples are shown in orange and red. Abbreviations: doses, vaccine doses; PC, post-COVID. Images were produced with GraphPad Prism V9 and mounted with Adobe Phothoshop CS6.

1.2 Supplementary Tables

Supplementary Table 1. Vaccination data (regarding Fig. 1)					
	**Comirnaty (Pfizer)**	**Spikevax (Moderna)**	**Janssen (Janssen)**	**Vaxzevria (AstraZeneca)**	**Total**
**COVID 2 doses. Total vaccinated n= 10. Date and vaccine type known, sub-total n= 5**					
1st	4 (80)	0	0	1 (20)	5
2nd	3 (60)	2 (40)	0	0	5
Days D1- D2/ IQR	135/ 153				
Pattern 2 doses (n= 5)					
Inf-V-V	1 (20)				
Inf-V-V-Inf	3 (20)				
V-V-inf	1 (60)				
**COVID 3 doses. Total vaccinated n= 20. Date and vaccine type known, sub-total n= 18**					
1st	9 (50)	2 (11)	0	7 (39)	18
2nd	11 (61)	3 (17)	0	4 (22)	18
3rd	6 (33)	12 (67)	0	0	18
Days D1- D2/ IQR	63/ 71				
Days D2- D3/ IQR	199/ 25				
Pattern 3 doses (n= 18)					
V-V-V-Inf	15 (83)				
Inf-V-V-V	3 (17)				
**Post-COVID 2 doses. Total vaccinated n= 47. Date and vaccine type known, sub-total n= 22**					
1st	18 (82)	1 (4)	2 (9)	1 (4)	22
2nd	14 (64)	8 (36)	0	0	22
Days D1- D2/ IQR	152/ 198				
Pattern 2 doses (most frequent)					
Inf-V-V	12				
Inf-V-V-Inf	4				
V-V-inf	2				
**Post-COVID 3 doses. Total vaccinated n= 21. Date and vaccine type known, sub-total n= 6**					
1st	5 (83)	0	0	1 (17)	6
2nd	5 (83)	1 (17)	0	0	6
3rd	4 (67)	2 (34)	0	0	6
Days D1- D2 / IQR	74/ 94				
Days D2- D3/ IQR	196/ 20				
Pattern 3 doses (n= 6)					
Inf-V-V-V	3 (50)				
Inf-V-V-V-Inf	3 (50)				
**Post-COVID 1 dose. Total vaccinated n= 17. Date and vaccine type known, sub-total n= 9**					
	7 (78)	1 (11)	0	1 (11)	9
Pattern 1 dose (most frequent)					
Inf-V-Inf	6 (86)				
**Post-COVID. Total n= 19 (0 doses)**					
% are shown in parenthesis. Abbreviations: D, dose, IQR: interquartile range, Inf, infection, V, vaccine.					

Supplementary Table 2. Spearman correlations (Fig. 2E-G)

		COVID					Post- COVID				
		**IgG N**	**IgG FL-S**	**IgG1 FL-S**	**IgG2 FL-S**	**IgG4 FL-S**	**IgG N**	**IgG FL-S**	**IgG1 FL-S**	**IgG2 FL-S**	**IgG4 FL-S**
**IgG N**	r_s	1	0.2673	0.0898	-0.1500	-0.1193	1	0.26820 1932	0.24486 9545	0.03273 2753	-0.1525
	p	0	0.1532	0.6367	0.4459	0.5451	0	**0.0059**	**0.0122**	0.7415	0.1221
**IgG FL-S**	r_s	0.26733	1	0.5888	-0.0492	-0.4438	0.2682	1	0.9068	0.3381	-0.1315
	p	0.1532	0	**0.0006**	0.8034	**0.0179**	**0.0059**	0	**2.29 E-40**	**0.0004**	0.1832
**IgG1 FL-S**	r_s	0.0898	0.5888	1	0.0925	-0.2134	0.2448	0.9068	1	0.5416	-0.0253
	p	0.6367	**0.0006**	0	0.6396	0.2754	**0.0122**	**2.297 E-40**	0	**2.8979 E-09**	0.7986
**IgG2 FL-S**	r_s	- 0.1500	-0.0492	0.0925	1	0.6759	0.0327	0.3381	0.5416	1	0.4614
	p	0.4459	0.8034	0.6396	0	**7.8795 E-05**	0.7415	**0.0004**	**2.8979 E-09**	0	**8.1991 E-07**
**IgG4 FL-S**	r_s	-0.1193	-0.4438	-0.2134	0.6759	1	-0.1525	-0.1315	-0.0253	0.46148 409	1
	p	0.5451	**0.0179**	0.2754	**7.879 E-05**	0	0.1221	0.1832	0.7986	**8.1991 E-07**	0

		COVID				Post-COVID			
		**IgG N**	**IgG1 RBD**	**IgG2 RBD**	**IgG4 RBD**	**IgG N**	**IgG1 RBD**	**IgG2 RBD**	**IgG4 RBD**
**IgG N**	r_s	1	-0.2041	-0.4292	-0.4292	1	0.23978 8325	-0.0333	0.01091 7869
	p	0	0.2973	**0.0226**	**0.0226**	0	**0.0142**	0.7367	0.9124
**IgG1 RBD**	r_s	-0.2041	1	0.2337	0.1674	0.2397	1	0.3885	0.5306
	p	0.2973	0	0.2313	0.3942	**0.0142**	0	**4.58243 E-05**	**6.85214 E-09**
**IgG2 RBD**	r_s	-0.4292	0.2337	1	0.8949	-0.0333	0.3885	1	0.7316
	p	**0.02265**	0.23130	0	**1.329 E-10**	0.73671	**4.582 E-05**	0	**5.6611 E-19**
**IgG4 RBD**	r_s	-0.4292	0.1674	0.8949	1	0.0109	0.5306	0.7316	1
	p	0.0226	0.3942	**1.329 E-10**	0	0.9124	**6.85214 E-09**	**5.661 E-19**	0

Supplementary Table 2 cont.

		COVID						Post-COVID					
		**IgG1 FL-S**	**IgG2 FL-S**	**IgG4 FL-S**	**IgG1 RBD**	**IgG2 RBD**	**IgG4 RBD**	**IgG1 FL-S**	**IgG2 FL-S**	**IgG4 FL-S**	**IgG1 RBD**	**IgG2 RBD**	**IgG4 RBD**
**IgG1 FL-S**	r_s	1	0.0925	-0.2134	-0.2813	-0.2917	-0.2172	1	0.5416	-0.02531	0.7303	0.3070	0.4519
	p	0	0.6396	0.2754	0.1469	0.1319	0.2666	0	**2.8979 E-09**	0.7986	**6.9318 E-19**	**0.0015**	**1.4621 E-06**
**IgG2 FL-S**	r_s	0.092	1	0.6759	0.3990	0.4504	0.3486	0.5416	1	0.4614	0.4917	0.3028	0.2512
	p	0.6396	0	**7.8795 E-05**	0.0354	0.0161	0.0690	**2.8979 E-09**	0	**8.1991 E-07**	**1.1546 E-07**	0.0017	0.0100
**IgG4 FL-S**	r_s	-0.2134	0.6759	1	0.2736	0.6902	0.7033	-0.0253	0.4614	1	0.1168	0.4462	0.4431
	p	0.2754	**7.8795 E-05**	0	0.1587	**4.8172 E-05**	**2.9835 E-05**	0.7986	**8.1991 E-07**	0	0.2374	**2.0615 E-06**	**2.469E-06**
**IgG1 RBD**	r_s	-0.2813	0.3990	0.2736	1	0.2337	0.1674	0.7303	0.4917	0.1168	1	0.3871	0.5307
	p	0.1469	0.0354	0.1587	0	0.2313	0.3942	**6.9318 E-19**	**1.1546 E-07**	0.23743	0	**4.9066 E-05**	**6.8389 E-09**
**IgG2 RBD**	r_s	-0.2917	0.4504	0.6902	0.2337	1	0.8949	0.3070	0.3028	0.4462	0.3871	1	0.7319
	p	0.1319	0.0161	**4.8172 E-05**	0.2313	0	**1.3295 E-10**	**0.0015**	**0.0017**	**2.0615 E-06**	**4.9066 E-05**	0	**5.3849 E-19**
**IgG4 RBD**	r_s	-0.2172	0.3486	0.7033	0.16748	0.8949	1	0.4519	0.2512	0.44312	0.5307	0.7319	1
	p	0.2666	0.0690	**2.9835 E-05**	0.3942	**1.3295 E-10**	0	**1.4621 E-06**	**0.0100**	**2.469 E-06**	**6.8389 E-09**	**5.3849 E-19**	0

Supplementary Table 3. Reinfections for samples included in Fig 3												
	COVID 2 doses		COVID 3 doses		Post-COVID 0 doses		Post-COVID 1 dose		Post-COVID 2 doses		Post-COVID 3 doses	
Total	9	%	21	%	19	%	17	%	47	%	21	%
No reinfected	7	78	17	81	8	42	3	18	29	62	11	52
Reinfected	2	22	4	19	11	58	13	76	15	32	10	48
2 reinfections	0	0	0	0	0	0	1	6	3	6	0	0

Supplementary Table 4.		
	Post-COVID Infected 2020 and vaccinated with 2 and 3 doses	
	No Reinfected	Reinfected (6 months)
N (total)	34	18
Age Average (IQR)	50 (9)	48 (8)
Woman n (%)	31 (91)	17 (94)
BMI (IQR)/ Obese n (%)	26 (9)/ 6 (18)	25 (7)/ 3 (17)
Previous disease n (%)	17 (50)	12 (67)
Hospitalization / ICU	13 (38)/ 2 (6)	2 (11)/ 0 (0)
COVID pneumonia	19 (56)	4 (22)
Months from primo infection to sample (IQR)	28 (1)	27 (2)
Months from reinfection to sample (IQR)		3.2 (4)
Mean date vaccination<br>(First, second, third dose)	May 28, 2021 (n= 18, 17 Pfizer, 1 Moderna)<br>Oct. 15. 29, 2021 (n= 18, 12 Pfizer, 6 Moderna)<br>March 26, 2022 (n= 4, 3 Pfizer, 1 Moderna)	
